# Deformity Correction in an Adult With Hypophosphatemic Rickets

**DOI:** 10.7759/cureus.92503

**Published:** 2025-09-16

**Authors:** Asim Mohabbat, Samirah S Alharbi, Mashael M Muwanis

**Affiliations:** 1 Orthopedic Surgery, Ministry of the National Guard – Health Affairs, Prince Mohammed Bin Abdulaziz Hospital, Medina, SAU; 2 Orthopedics, College of Medicine, Taibah University, Medina, SAU

**Keywords:** deformity, osteotomy, tibial bowing, vitamin d-resistant hypophosphatemic rickets, x-linked disorder

## Abstract

Vitamin D-resistant hypophosphatemic rickets is an X-linked genetic disorder that presents with varying degrees of bone deformities that usually involve the tibia and femur. The deformities affect the patient’s quality of life and restrict their ability to ambulate, resulting in significant psychological distress. In this report, we present an adult case of hypophosphatemic rickets with severe bilateral tibial bowing, treated with multiple osteotomies and intramedullary nail fixation, resulting in marked improvement in ambulation.

## Introduction

Hypophosphatemic rickets (HR) is a genetic disorder, with the X-linked hereditary type being the most prevalent, accounting for approximately 80% of HR cases [1]. The clinical presentation typically includes lower limb deformities, such as bowing of the tibia and femur, bilateral genu varum or valgum, often combined with tibial torsion, and disproportionately short stature. These manifestations generally become apparent within the first two years of life [2].

The primary treatment for HR involves high doses of 1,25-dihydroxy vitamin D [1,25 (OH)₂D] either alone or in combination with oral phosphate supplementation. For severe deformities and pathological fractures, surgical interventions, including osteotomies, plating, and intramedullary fixation, are considered [3-5]. However, these surgical procedures are typically performed during childhood. This report presents the case of a 21-year-old male with neglected HR and progressive deformity, who underwent multiple osteotomies and intramedullary nail fixation, achieving promising outcomes.

## Case presentation

A 21-year-old male patient, a known case of HR, presented with a positive family history of the condition affecting his mother, four sisters, and three brothers. His past medical history included multiple bilateral tibial and femoral fractures at various ages, some of which were treated surgically while others were managed with casting and immobilization.

**Figure 1:**
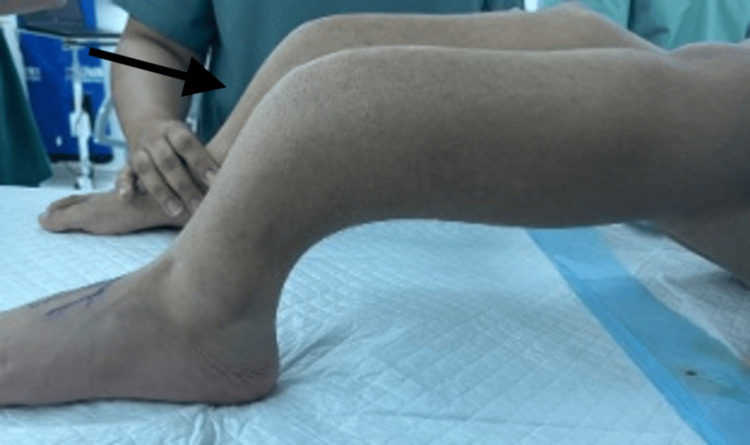
Preoperative patient photograph showing bilateral tibial bowing deformity (arrow)

The patient had been walking independently until the age of 12, when he sustained a left tibial shaft fracture treated with a cast. Following this, he developed progressive bilateral tibial bowing, which eventually prevented him from walking. He presented to our clinic complaining of severe bilateral tibial bowing and inability to walk (Figure 1).

**Figure 2:**
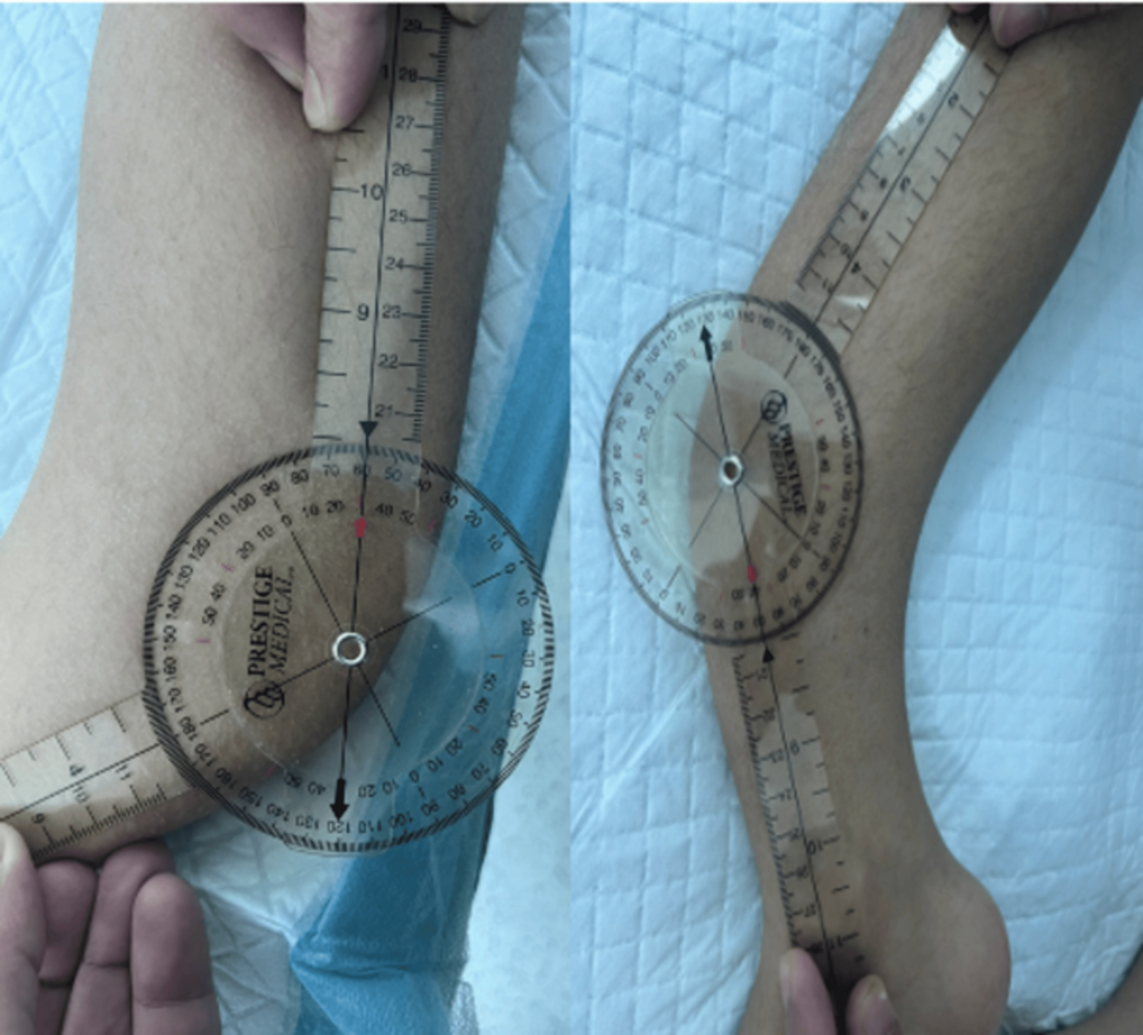
Goniometer measurements (the right side is the right leg, and the left side is the left leg) The goniometer showing 50-degree anterior bowing of the right tibia and 60 degrees on the left tibia.

Upon examination, the patient was mobilizing by crawling for short distances using his hands and relying on a wheelchair for longer distances. He exhibited bilateral anterior tibial midshaft bowing, with an angle of approximately 60 degrees on the left leg and 50 degrees on the right leg, measured using a goniometer (Figure 2).

Quadriceps muscle strength was normal bilaterally. The range of motion in his right knee was within normal limits, with 5 degrees of extension and 150 degrees of flexion. However, the left knee range of motion was restricted, with 0 degrees of extension and 70 degrees of flexion. This limitation was attributed to a previous femoral shaft fracture, which was treated surgically (where plate and screw fixation had been performed), and post-operatively, he started noticing his range-of-motion limitation. The patient also demonstrated bilateral tight Achilles tendons, with ankle dorsiflexion of -5 and plantar flexion of 50 degrees bilaterally.

Imaging and preoperative planning

**Figure 3:**
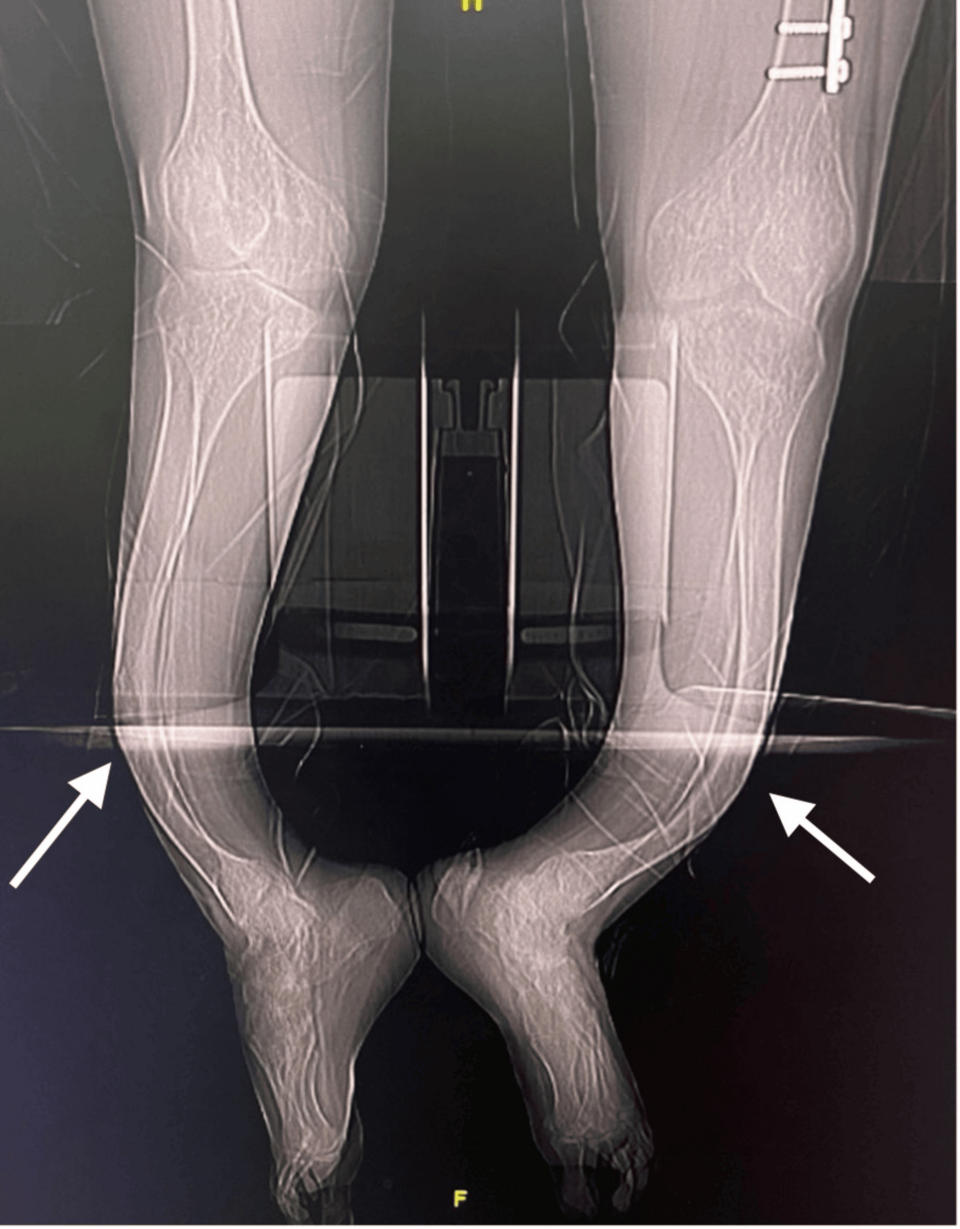
Preoperative CT scan showing severe bilateral anterior bowing of the bilateral tibia (arrow)

Preoperative bilateral full-length tibia anteroposterior and lateral X-ray views were obtained. The angle of deformity at the center of rotation and angulation (CORA) was measured to assist with osteotomy planning. Additionally, the length and diameter of the medullary canal were measured to determine the appropriate size of the intramedullary nail. The preoperative X-ray images were very difficult to obtain due to his deformity; thus, CT scanography images were obtained, as shown in Figure 3.

Surgical technique

The patient was positioned supine on a radiolucent table. The procedure began with removal of previous plate and screws in his left femur to prevent stress riser in the future. For his deformity correction, we started with the right tibia. An anteromedial approach was taken, centered at the level of the CORA. Layered dissection was performed down to the bone, followed by subperiosteal dissection around the CORA. Due to bone fragility and to prevent fracture propagation, two osteotomies were performed using a "shish-kebab" technique. A bone fragment was removed and reamed on a side table with a manual rigid reamer, starting from size 6 and progressing to the appropriate nail size. Retrograde reaming was performed from the osteotomy site to identify the proper nail entry location. The anterior knee approach was then utilized, accessing the entry point through the patellar tendon. A guide wire was inserted through both the proximal and distal tibial segments. Reaming was carried out in an antegrade direction until the canal was prepared for nail insertion. The intramedullary locked tibial nail was inserted through the proximal segment to the osteotomy site, passing through the bone fragment along the predetermined markings and into the distal tibial segment. Correct rotational alignment of the tibia was ensured using pre-osteotomy markings and by referencing the foot and femoral condyle positions. Two proximal and one distal interlocking screws were then inserted.

The same procedure was performed on the left tibia; however, three osteotomies were required to correct the deformity. Additionally, bilateral Achilles tendon Z-lengthening was performed to achieve 10 degrees of ankle dorsiflexion bilaterally.

**Figure 4:**
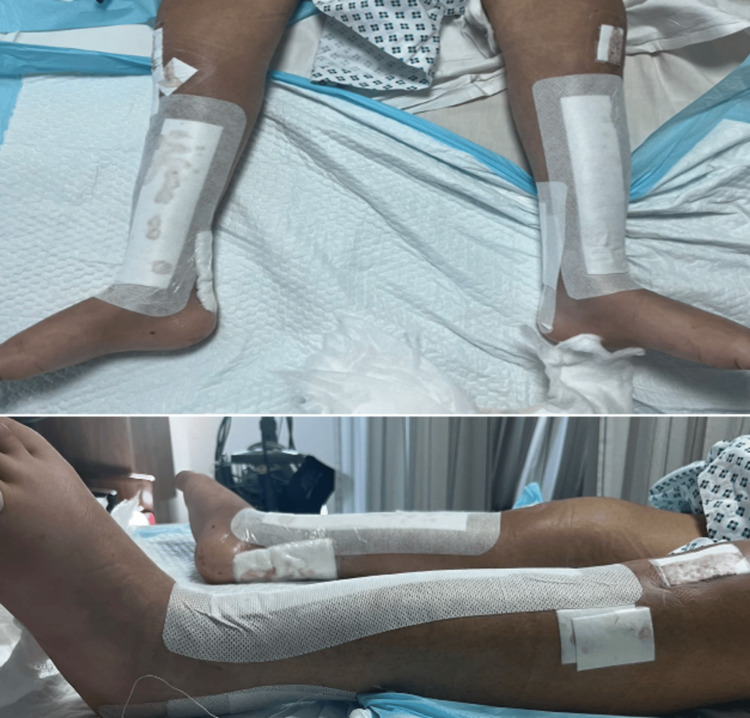
Postoperative patient photograph Postoperative clinical photographs showing both lower limbs with surgical dressings in place following multiple tibial osteotomies and intramedullary nail fixation for the correction of bilateral tibial bowing

**Figure 5:**
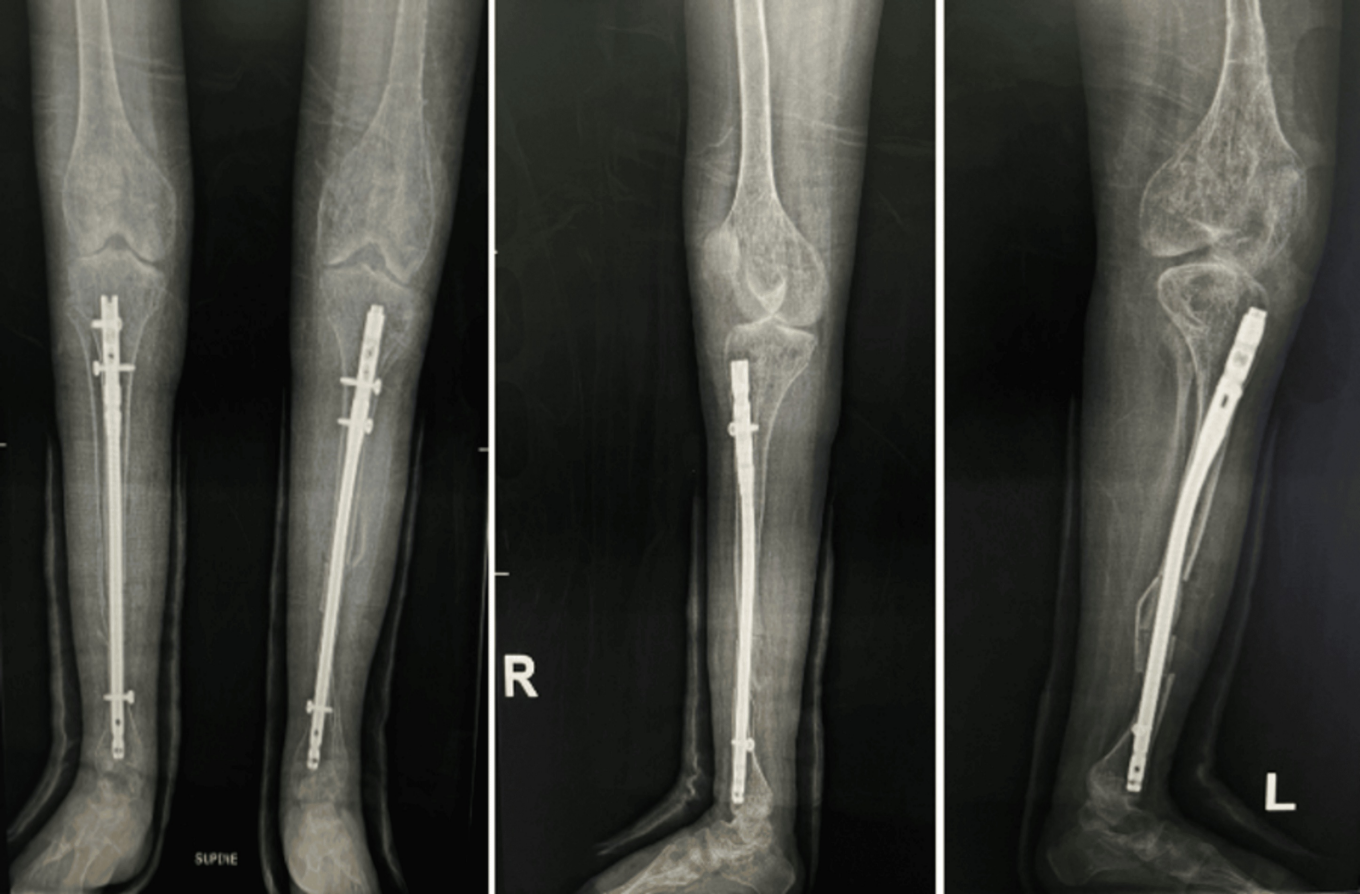
Postoperative patient X-rays Postoperative radiographs of both tibiae demonstrating intramedullary nail fixation following multiple corrective osteotomies for bilateral tibial bowing deformity. Anteroposterior and lateral views show satisfactory alignment and stable fixation of the right and left tibiae.

Postoperatively, the patient was placed in bilateral below-knee walking casts with the ankles positioned in neutral dorsiflexion to ensure proper healing of the Achilles tendons. Postoperative photographs and X-ray images are shown in Figures 4, 5. The patient tolerated the surgery well without any complications.

**Figure 6:**
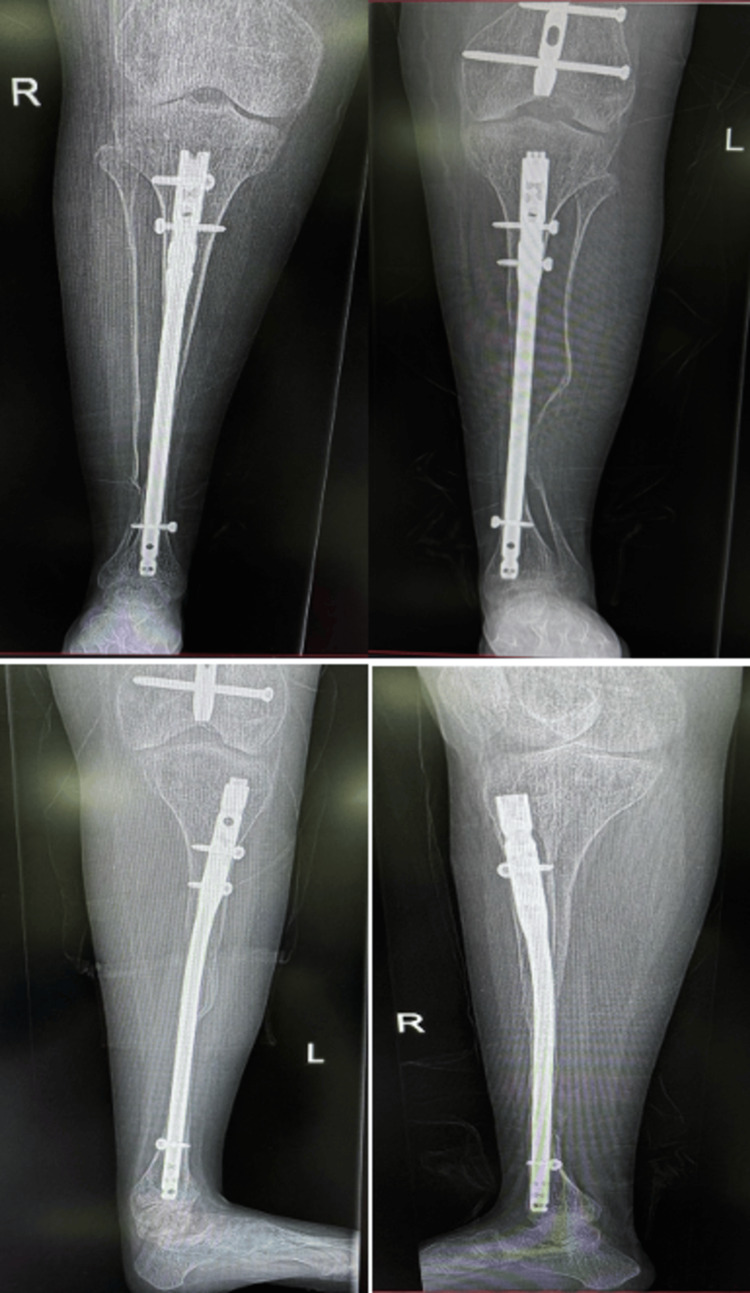
X-rays six months postoperatively Six-month follow-up radiographs of both tibiae in anteroposterior and lateral views showing maintained alignment and stable intramedullary nail fixation following corrective osteotomies. Evidence of satisfactory healing is noted without signs of implant loosening or displacement.

**Figure 7:**
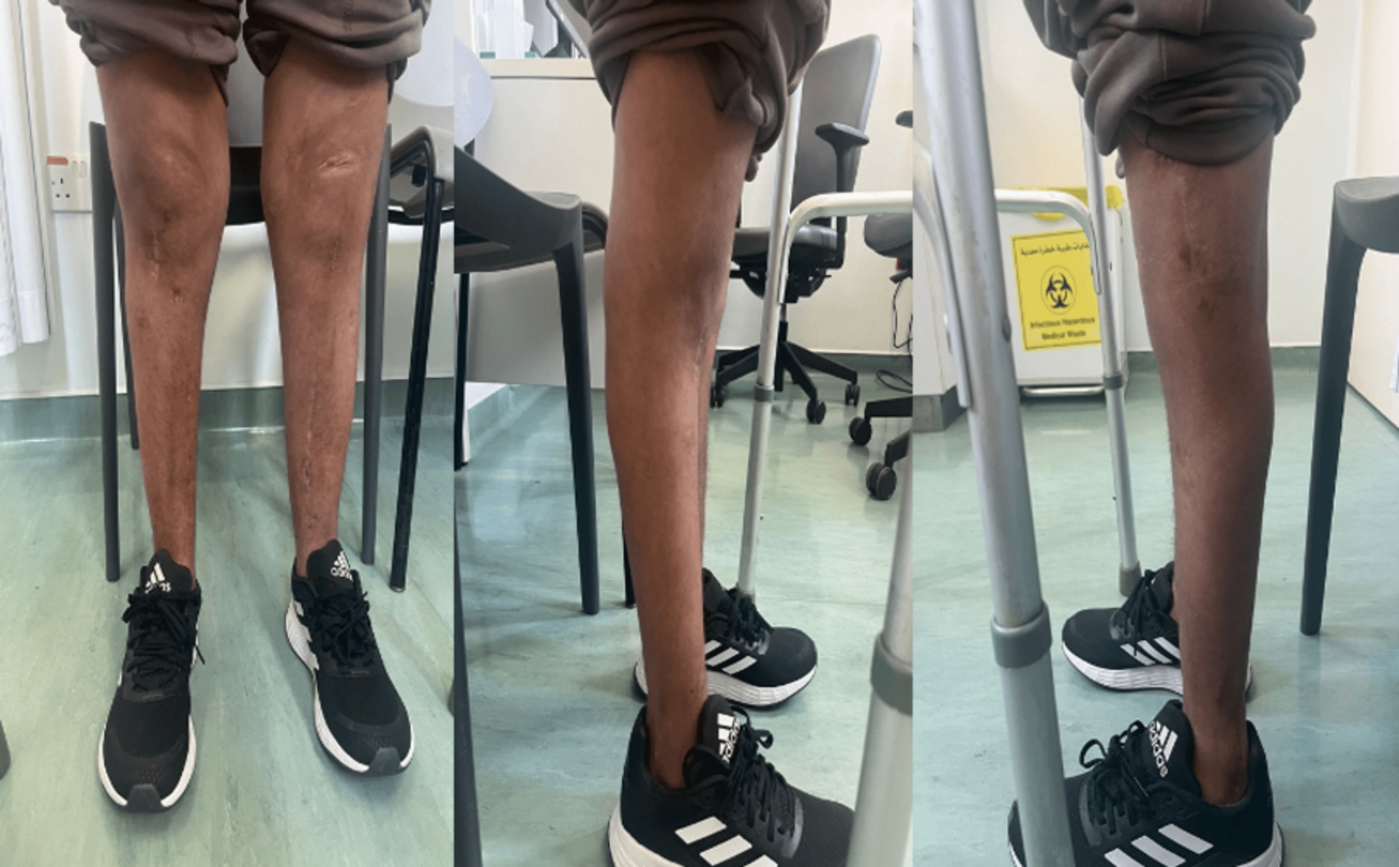
Clinical photograph six months postoperatively Clinical photographs showing the lower limbs six months post-surgery. The images demonstrate well-aligned tibiae with healed surgical scars and no signs of infection or inflammation.

During the postoperative period, the patient's clinical condition remained optimal. He was allowed to ambulate using a walking frame starting on the second postoperative day. At his most recent follow-up, six months later, the patient was mobilizing effectively, walking unassisted at home, and using a walker for long distances. Latest X-rays are shown in Figure 6, and the latest clinical photographs are shown in Figure 7.

## Discussion

Vitamin D-resistant hypophosphatemic rickets (VDHR), an X-linked genetic disorder first described by Albright et al. in 1937 [1], is the most common form of rickets encountered in orthopedic practice. The clinical presentation in adults includes a wide spectrum of musculoskeletal manifestations, such as short stature, osteomalacia, bone pain, pseudofractures, fragility fractures, bone deformities, degenerative joint disease, early osteoarthritis, joint stiffness, reduced joint mobility, hearing loss, and extra-osseous calcifications, including enthesophytes and spinal stenosis. Additionally, patients may experience muscle pain, weakness, and dental abnormalities [6-8]. In our patient, the most prominent clinical findings were severe bilateral anterior tibial midshaft bowing and an inability to walk.

While early intervention with high-dose vitamin D therapy during childhood can often prevent or reduce long bone deformities and promote pseudo fracture healing [9], many adults present with significant, established deformities requiring surgical correction, even with adequate or delayed medical management. Various surgical techniques have been described to address bowing deformities of the lower extremities in VDHR, including multiple osteotomies with intramedullary nailing [3,5,10], plate fixation [3-5,11], epiphysiodesis [4,12], and external fixation [13] .

Multiple osteotomies with intramedullary nailing have proven to be particularly effective for correcting severe femoral and tibial shaft deformities [3,4]. This technique, first reported by Sofield and Millar in 1959 [10] for children with congenital pseudarthrosis, osteogenesis imperfecta, or fibrous dysplasia, demonstrated a low complication rate. Of the six patients with VDHR included in their study, there were no reported cases of non-union or vascular complications, with only three major complications overall: two cases of serious infection and one nerve injury. In our case, the patient tolerated the procedure well, experiencing no complications. He began ambulating with a walking frame on the second postoperative day, and, at the six-month follow-up, he achieved union of his osteotomies, no infection was recorded, and he was walking unassisted.

A key distinction between VDHR in children and adults lies in the potential for deformity correction through medication. While children often respond favorably to medical management, adults typically present with fixed deformities that are unresponsive to pharmacological therapy and therefore require surgical intervention. In children with severe leg deformities, surgical treatment may be necessary; however, it is generally recommended to delay surgery until after growth plate closure. This approach minimizes the risk of recurrence, which is more common in children than in adults [14].

To our knowledge, published reports on the surgical management of severe bilateral tibial deformities in adults with VDHR are scarce. Most studies focus on pediatric patients, in whom medical therapy or corrective surgery after skeletal maturity are the main approaches [3-5,9]. Even among adult cases described in the literature, unilateral deformities or milder presentations are more commonly reported, and outcomes have been mixed due to complications such as recurrence, infection, or delayed union [10-13]. In contrast, our patient presented with severe, symmetrical tibial bowing that rendered him unable to walk, yet achieved uneventful bone healing and independent ambulation within six months following one-stage multiple osteotomies and intramedullary nailing. This case, therefore, demonstrates both the feasibility and durability of this technique in adult patients with advanced VDHR, emphasizing that meaningful functional recovery can be achieved even in the setting of longstanding, rigid deformities unresponsive to medical therapy.

The primary goals of surgical intervention for limb deformities secondary to VDHR are deformity correction, restoration of the lower limb load line, improvement of lower limb function, and enhancement of the patient’s quality of life. In this case, the surgical procedure was aimed at correcting the severe bowing of both tibiae and improving the patient’s ambulation. Postoperatively, the patient demonstrated significant improvement.

## Conclusions

This case highlights the challenges and effective management of severe bilateral tibial deformities in an adult with HR. Despite the long-standing inability to walk and significant functional limitations, the combination of medical therapy and single-stage surgical correction, involving multiple osteotomies and intramedullary nail fixation, resulted in remarkable clinical improvement. Early ambulation with a walking frame by the second postoperative day underscores the success of the intervention in restoring mobility and enhancing the patient’s quality of life. This report emphasizes the importance of a multidisciplinary approach in managing complex deformities associated with HR, providing valuable insights for clinicians dealing with similar cases.
